# Authoritative Parenting Is Associated with Healthier Lifestyle Patterns in University Students

**DOI:** 10.3390/healthcare14111521

**Published:** 2026-05-30

**Authors:** Maja Strauss, Barbara Cussigh, Leona Cilar Budler

**Affiliations:** Faculty of Health Sciences, University of Maribor, 2000 Maribor, Sloveniabarbara.cussigh@um.si (B.C.)

**Keywords:** parenting styles, authoritative parenting, health-promoting lifestyle, university students, health behaviors, Health-Promoting Lifestyle Profile II, young adults, lifestyle behaviors

## Abstract

**Background:** Health-promoting lifestyle behaviors established during young adulthood play a crucial role in shaping long-term physical and mental health outcomes, including the risk of chronic disease, psychological well-being, and quality of life. Parenting styles represent an important psychosocial factor that may be associated with health-related behaviors; however, evidence regarding their association with multidimensional health-promoting lifestyles among university students remains limited. **Methods:** A cross-sectional study was conducted among 700 university students. Parenting styles (authoritative, authoritarian, and permissive) were assessed using validated self-report measures. Health-promoting lifestyle behaviors were measured with the Health-Promoting Lifestyle Profile II (HPLP II), including six subscales: Health Responsibility, Physical Activity, Nutrition, Spiritual Growth, Interpersonal Relations, and Stress Management, as well as the overall HPLP II score. Multiple linear regression analyses were performed to examine associations between parenting styles and each HPLP II subscale and the total score. **Results:** All regression models were statistically significant (*p* < 0.001), explaining between 5.2% and 13.5% of variance across HPLP II subscales and 11.8% of variance in the total score. Authoritative parenting was significantly positively associated all health-promoting lifestyle domains (β = 0.22–0.33, *p* < 0.001), including physical activity, interpersonal relations, stress management, and overall health-promoting lifestyle. Permissive parenting was negatively associated with several domains, particularly physical activity, interpersonal relations, stress management, and the total HPLP II score (β = −0.07 to −0.12, *p* < 0.05). Authoritarian parenting showed weaker and more selective negative associations, most notably with nutrition and stress management. **Conclusions:** Parenting styles are significantly associated with health-promoting lifestyle behaviors among university students. Authoritative parenting was consistently associated with more favorable health-promoting lifestyle patterns across multiple domains, whereas permissive and authoritarian parenting may be linked to less favorable health behaviors. These findings suggest that perceived parenting styles are associated with health-related behaviors among university students.

## 1. Introduction

Establishing a healthy lifestyle during adolescence and early adulthood serves as a critical protective factor for long-term physical and mental health [1]. Students who experience significant developmental changes frequently adopt behavioural patterns related to physical activity, nutrition, stress management, and interpersonal relationships that persist into adulthood [2,3,4]. This period is marked by increased autonomy, evolving social roles, and heightened exposure to environmental and behavioural risks, all of which substantially influence the formation of lifestyle habits [3,5]. Adopting a healthy lifestyle enhances quality of life and psychological well-being and reduces the risk of chronic diseases [6,7]. Recent data from 2022 show that over 80% of adolescents do not meet the WHO’s recommendations for physical activity [1], healthy eating, and effective stress management, thereby increasing the risk of chronic disease and poorer mental health in later life [8,9].

The family environment represents an important contextual influence on health-promoting behaviour, particularly through parenting styles, which are defined by varying degrees of warmth (responsiveness) and behavioural control [10,11,12]. Authoritative, authoritarian, and permissive parenting styles differ in their levels of warmth and control. Parental experiences and perceived parenting patterns may remain relevant during emerging adulthood, shaping both psychological and behavioural processes [10,11]. Parenting style has been associated with self-regulation, coping strategies, and decision-making, all of which are directly linked to the health and well-being of young adults [10].

Recent research demonstrates that authoritative parenting, characterized by high levels of support and structure, is associated with positive outcomes such as enhanced psychological well-being, improved self-regulation, and healthier behaviours [10,12,13]. In contrast, authoritarian and permissive parenting styles are linked to less favourable outcomes, including increased stress, maladaptive coping strategies, and a higher risk of unhealthy behaviours [11,12,13]. Authoritative parenting has been associated with more favorable outcomes among young adults by promoting positive social and behavioural patterns [10,12].

Although there is substantial evidence regarding the impact of parenting styles, most research focuses on isolated outcomes such as mental health, substance use, or specific risk behaviours [14]. Few studies investigate the holistic concept of a health-promoting lifestyle, which includes physical activity, nutrition, interpersonal relationships, and stress management [15,16]. Evidence from university student populations suggests that health-promoting behaviors are influenced by multiple demographic, psychosocial, and contextual factors, further supporting the need to examine developmental influences such as parenting styles [17].

This study investigates the association between parenting styles and health-promoting behaviours, focusing on six lifestyle dimensions: health responsibility, physical activity, nutrition, spiritual growth, interpersonal relationships, and stress management. This approach clarifies the influence of the family environment on health during adolescence and emerging adulthood.

## 2. Methods

### 2.1. Study Design

A cross-sectional observational study was conducted to examine the association between perceived parenting styles and health-promoting lifestyle behaviors among university students. The study followed a quantitative research design using an online self-administered questionnaire.

### 2.2. Sample and Procedure

The study sample consisted of undergraduate and graduate students enrolled at various faculties of a public university. Data were collected using an online survey distributed via institutional communication channels and social media platforms to maximize study visibility and reach among eligible students. To encourage participation, the survey was designed as an anonymous online questionnaire, allowing convenient access and completion at participants’ preferred time while minimizing respondent burden. Participation was entirely voluntary. A total of 701 questionnaires were returned; after excluding cases with missing data on key variables, 700 participants were included in the final analyses.

### 2.3. Instruments

Perceived parenting styles were assessed using a brief parenting style measure adapted from Robinson et al. [18], based on Baumrind’s three parenting style framework (authoritative, authoritarian, and permissive). The brief version was selected to enable efficient assessment of perceived parenting experiences within a broader survey while minimizing respondent burden, an important consideration in large student-based questionnaire studies. Although shorter instruments may provide less detailed construct coverage than full parenting inventories, they have been used in prior research to capture global parenting style dimensions in a pragmatic and theoretically grounded manner. The instrument consisted of nine items, with three items representing each parenting style dimension. Participants rated the extent to which each statement reflected their general parental upbringing experience using a 5-point Likert scale ranging from 1 (very untrue/uncharacteristic) to 5 (very true/characteristic). Higher scores indicated greater perceived presence of the respective parenting style. Participants evaluated parental behavior in general rather than mothers and fathers separately. Internal consistency in the present sample was acceptable for authoritarian parenting (Cronbach’s α = 0.75), modest for authoritative parenting (α = 0.64), and low for permissive parenting (α = 0.48). The relatively low internal consistency of the permissive parenting subscale suggests limited measurement precision for this construct and should be considered when interpreting findings related to permissive parenting.

Health-promoting lifestyle behaviors were measured using the Health-Promoting Lifestyle Profile II (HPLP II) [19]. The instrument assesses the frequency of health-promoting behaviors across six subscales: Health Responsibility (HR), Physical Activity (PA), Nutrition (NU), Spiritual Growth (SG), Interpersonal Relations (IR), and Stress Management (SM). Responses were recorded on a 4-point Likert scale ranging from 1 (never) to 4 (routinely). Subscale scores were calculated as the mean of the corresponding items, with higher scores indicating more frequent engagement in health-promoting behaviors. The overall HPLP II total score was calculated as the mean of all available items.

### 2.4. Data Analysis

Data were analyzed using IBM SPSS Statistics version 30.0. Descriptive statistics, including means, standard deviations, observed ranges, and internal consistency reliability coefficients, were calculated for all main study variables. Pearson zero-order correlations were computed to examine bivariate associations among parenting styles and HPLP II outcomes. Multiple linear regression analyses were then performed to examine associations between perceived parenting styles and each HPLP II subscale as well as the total HPLP II score. All regression models were adjusted for gender, age, living arrangement, employment status, and chronic health condition. Gender and employment status were entered as categorical covariates, age as a continuous covariate, and living arrangement and chronic health condition as binary covariates.

Prior to interpreting the regression models, assumptions of multiple linear regression were evaluated. Multicollinearity was assessed using variance inflation factors (VIF) and tolerance values. Independence of residuals was examined using the Durbin–Watson statistic, and influential observations were assessed using Cook’s distance. Residual distributions and model diagnostics were inspected to evaluate linearity, normality, and homoscedasticity. Because heteroscedasticity was indicated in some models, robust HC3 standard errors were used in the final adjusted regression models. Given the number of statistical comparisons, the Benjamini–Hochberg false discovery rate (FDR) correction was applied to the main parenting style coefficients. Statistical significance was set at *p* < 0.05.

## 3. Results

Table 1 presents the main characteristics of the participants included in the analysis. The sample consisted of 701 students, the majority of whom were female and enrolled in full-time study programs. The mean age of the participants was 20.41 years (SD = 3.23).

Table 2 presents descriptive statistics, observed score ranges, and internal consistency reliability coefficients for the parenting style variables and health-promoting lifestyle measures included in the study.

Zero-order correlations among the main study variables are presented in Table 3. Perceived authoritative parenting was positively correlated with all health-promoting lifestyle domains and the total HPLP II score. In contrast, perceived authoritarian and permissive parenting showed weaker and predominantly negative associations with several lifestyle dimensions. Correlations among parenting style variables were small to moderate, suggesting conceptual relatedness without problematic overlap.

To further examine these associations, adjusted multiple linear regression analyses were conducted for each HPLP II domain and the total HPLP II score (Table 4). All models were adjusted for gender, age, living arrangement, employment status, and chronic health condition.

Prior to model interpretation, regression assumptions were evaluated. Multicollinearity diagnostics indicated no problematic overlap among predictors, with variance inflation factor (VIF) values ranging from 1.04 to 1.19 for the parenting style variables and remaining below accepted thresholds across all adjusted models. Durbin–Watson statistics ranged from 1.88 to 2.00, indicating no evidence of residual autocorrelation. No observation exceeded Cook’s distance of 1.0. Because heteroscedasticity was detected in some models, robust HC3 standard errors were used in the final analyses. To reduce the risk of inflated Type I error due to multiple testing, the Benjamini–Hochberg false discovery rate (FDR) correction was applied to the main parenting style coefficients.

After adjustment and FDR correction, perceived authoritative parenting remained consistently positively associated with all HPLP II domains and the total HPLP II score, with standardized coefficients ranging from β = 0.156 for nutrition to β = 0.309 for interpersonal relations (all *p* < 0.001). Perceived permissive parenting showed weaker negative associations with physical activity, nutrition, and the total HPLP II score, whereas associations with health responsibility, interpersonal relations, and stress management were no longer statistically significant. Perceived authoritarian parenting demonstrated selective negative associations with spiritual growth, interpersonal relations, and stress management, while its association with the total HPLP II score did not remain statistically significant.

A multiple linear regression analysis was performed to examine the association between parenting styles and the Health Responsibility (HR) subscale. The overall regression model was statistically significant, F(3, 696) = 15.35, *p* < 0.001, accounting for 6.2% of the variance in health responsibility (adjusted R^2^ = 0.058).

Authoritative parenting was significantly positively associated with health responsibility (B = 0.14, β = 0.23, *p* < 0.001), indicating that higher levels of authoritative parenting were associated with more frequent engagement in health-responsible behaviors. In contrast, permissive parenting showed a small but statistically significant negative association with health responsibility (B = −0.05, β = −0.08, *p* = 0.044). Authoritarian parenting was not significantly associated with health responsibility (*p* = 0.490) (Table 5).

A multiple linear regression analysis was conducted to examine the association between parenting styles and the Physical Activity subscale. The overall regression model was statistically significant, F(3, 696) = 30.11, *p* < 0.001, explaining 11.5% of the variance in physical activity (adjusted R^2^ = 0.111).

Perceived authoritative parenting was significantly positively associated with physical activity (B = 0.20, β = 0.31, *p* < 0.001), indicating that students who reported higher levels of authoritative parenting engaged more frequently in physical activity. In contrast, permissive parenting significantly negatively associated with physical activity (B = −0.06, β = −0.10, *p* = 0.008). Authoritarian parenting was not significantly associated with physical activity (*p* = 0.123) (Table 6).

A multiple linear regression analysis was conducted to examine the association between parenting styles and the Nutrition subscale. The overall regression model was statistically significant, F(3, 696) = 20.95, *p* < 0.001, explaining 8.3% of the variance in nutrition behaviors (adjusted R^2^ = 0.079).

Authoritative parenting was significantly positively associated with healthier nutrition behaviors (B = 0.15, β = 0.25, *p* < 0.001). In contrast, authoritarian parenting showed a small but statistically significant negative association with nutrition (B = −0.04, β = −0.09, *p* = 0.022). Permissive parenting was not significantly associated with nutrition behaviors (*p* = 0.446) (Table 7).

A multiple linear regression analysis was conducted to examine the association between parenting styles and the Spiritual Growth subscale. The overall regression model was statistically significant, F(3, 696) = 12.72, *p* < 0.001, explaining 5.2% of the variance in spiritual growth (adjusted R^2^ = 0.048).

Authoritative parenting was significantly positively associated with spiritual growth (B = 0.13, β = 0.22, *p* < 0.001), indicating that higher levels of authoritative parenting were associated with greater spiritual growth. Permissive parenting showed a marginally non-significant negative association with spiritual growth (B = −0.04, β = −0.07, *p* = 0.074). Authoritarian parenting was not significantly associated with spiritual growth (*p* = 0.572) (Table 8).

A multiple linear regression analysis was conducted to examine the association between parenting styles and the Interpersonal Relations subscale. The overall regression model was statistically significant, F(3, 696) = 36.09, *p* < 0.001, explaining 13.5% of the variance in interpersonal relations (adjusted R^2^ = 0.131).

Authoritative parenting was significantly positively associated with interpersonal relations (B = 0.21, β = 0.033, *p* < 0.001), indicating that higher levels of authoritative parenting were associated with better interpersonal functioning. In contrast, permissive parenting was significantly negatively associated with interpersonal relations (B = −0.08, β = −0.12, *p* < 0.001). Authoritarian parenting showed a marginally non-significant negative association with interpersonal relations (B = −0.03, β = −0.07, *p* = 0.072) (Table 9).

A multiple linear regression analysis was conducted to examine the association between parenting styles and the Stress Management subscale. The overall regression model was statistically significant, F(3, 696) = 20.98, *p* < 0.001, explaining 8.3% of the variance in stress management (adjusted R^2^ = 0.079).

Authoritative parenting was significantly positively associated with stress management (B = 0.16, β = 0.24, *p* < 0.001), indicating that higher levels of authoritative parenting were associated with more effective stress management behaviors. In contrast, both authoritarian (B = −0.04, β = −0.08, *p* = 0.038) and permissive parenting styles (B = −0.08, β = −0.11, *p* = 0.003) were significantly negatively associated with stress management (Table 10).

A multiple linear regression analysis was conducted to examine the association between parenting styles and the overall Health-Promoting Lifestyle Profile II (HPLP II) total score. The regression model was statistically significant, F(3, 696) = 31.18, *p* < 0.001, explaining 11.8% of the variance in overall health-promoting lifestyle behaviors (adjusted R^2^ = 0.115).

Authoritative parenting was significantly positively associated with the HPLP II total score (B = 0.16, β = 0.31, *p* < 0.001), indicating that higher levels of authoritative parenting were associated with more frequent engagement in health-promoting behaviors overall. Permissive parenting significantly negatively associated with the total HPLP II score (B = −0.05, β = −0.09, *p* = 0.010). Authoritarian parenting showed a marginally non-significant negative association with the overall health-promoting lifestyle score (B = −0.03, β = −0.07, *p* = 0.064) (Table 11).

After adjusting for gender, age, living arrangement, employment status, and chronic health condition, all regression models remained statistically significant (Table 12). Perceived authoritative parenting was consistently positively associated with all HPLP II domains and the total HPLP II score. Standardized regression coefficients ranged from β = 0.156 for nutrition to β = 0.309 for interpersonal relations, with all associations statistically significant at *p* < 0.001. Permissive parenting showed weaker negative associations with physical activity, nutrition, spiritual growth, and the total HPLP II score, whereas associations with health responsibility, interpersonal relations, and stress management were not statistically significant after adjustment. Authoritarian parenting was negatively associated with spiritual growth, interpersonal relations, and stress management, while its association with the total HPLP II score was marginal and did not reach conventional statistical significance.

## 4. Discussion

The aim of this study was to examine the association between perceived parenting styles and health-promoting lifestyle behaviors among university students. The findings indicate that perceived parenting styles appear to remain associated with health-related behaviors in young adulthood, as authoritative parenting showed consistent positive associations with all domains of health-promoting lifestyle behaviors, while permissive and authoritarian parenting demonstrated weaker and predominantly negative associations. These results support developmental theories suggesting that early family environments may be associated with longer-term behavioral and health patterns [20].

The strongest and most consistent finding was the positive association between authoritative parenting and all dimensions of the Health-Promoting Lifestyle Profile II. Students who perceived their parents as supportive, responsive, and appropriately demanding reported more frequent engagement in physical activity, healthier nutrition behaviors, better stress management, stronger interpersonal relationships, and higher overall engagement in health-promoting behaviors. This is consistent with developmental frameworks suggesting that authoritative parenting has been linked to autonomy, self-regulation, and competence, which are key determinants of healthy behavioral choices [20]. A parenting environment characterized by emotional support combined with clear expectations may be associated with the development of internal motivation and responsibility for personal health. Similar findings were reported by Alothman et al. [21], who identified significant associations between psychosocial and environmental factors and healthy lifestyle behaviors among university students, emphasizing the importance of supportive developmental contexts in shaping long-term health practices.

The findings also align with more recent research showing that authoritative parenting is associated with better psychological adjustment, healthier behavioral patterns, and higher well-being in adolescents and young adults [22,23]. Fung and Deng [24] further highlighted that authoritative parenting, even within culturally hybrid family environments, remains positively associated with emotional adaptation and balanced behavioral development among young people. One possible explanation is that authoritative parenting may be associated with self-efficacy development and adaptive coping strategies, which may later translate into more effective stress management and healthier daily routines. Additionally, such parenting may foster better communication skills and emotional security, which could explain the observed associations with interpersonal relations and psychosocial well-being [20].

Furthermore, research suggests that parenting styles may be associated with health behaviors through mechanisms such as behavioral modeling, emotional climate, and the development of self-regulation capacities, defined as the ability to regulate emotions, control impulses, plan behavior, and maintain goal-directed routines. These capacities have been proposed as plausible mechanisms that may help explain associations between early family experiences and later lifestyle choices [25]. Carbone et al. [26] similarly demonstrated that parenting style and parental emotion regulation indirectly influence young people’s mental health outcomes through self-efficacy and emotional regulation processes, supporting the interpretation that family emotional environments contribute to later adaptive functioning. These mechanisms may also explain why authoritative parenting in the present study was associated with both behavioral domains (physical activity and nutrition) and psychosocial domains (interpersonal relations and stress management).

In contrast, permissive parenting showed negative associations with several important health domains, particularly physical activity, interpersonal relations, stress management, and overall lifestyle behaviors. This may reflect the lower levels of behavioral structure and guidance typically associated with permissive parenting. Without consistent expectations or behavioral boundaries, young people may develop weaker self-discipline and health routines, which could negatively influence lifestyle behaviors requiring persistence and self-regulation [20]. Previous studies similarly suggest that permissive parenting may be associated with poorer behavioral regulation and increased risk behaviors compared to authoritative parenting [27].

Authoritarian parenting showed more limited but still relevant negative associations, particularly with nutrition and stress management. This finding may reflect the potential psychological costs of highly controlling parenting environments. Excessive control and lower emotional responsiveness may limit opportunities for the development of autonomous decision-making and adaptive coping strategies [28]. Students raised in such environments may either adopt externally regulated behaviors without internalizing health values or experience elevated stress levels, which may negatively influence their ability to regulate health behaviors effectively. Supporting this interpretation, Muniz et al. [29] found that maladaptive parenting styles were associated with impaired emotional regulation, stress-related coping, and higher engagement in risky health behaviors among university students, particularly alcohol-related problems mediated through stress mechanisms.

Although parenting styles explained a relatively modest proportion of variance in health-promoting behaviors, this finding is not unexpected. Health behaviors are complex and influenced by multiple determinants, including peer influences, socioeconomic factors, educational environments, personal motivation, and access to health resources [30]. Nevertheless, the consistent pattern of associations observed in this study suggests that parenting style may represent an important developmental background correlate of the formation of health-related behavioral tendencies. Comparable findings were observed by Uitumen and Tarkó [31], who reported that health-promoting behaviors among adolescents and young adults are shaped by multiple psychosocial and environmental influences, while family-related factors remain significant contributors to lifestyle development.

An important finding is that authoritative parenting was positively associated not only with behavioral domains such as physical activity and nutrition but also with psychosocial domains such as interpersonal relations and stress management. This supports the assumption that parenting style may influence broader psychosocial competencies that indirectly shape health behaviors. For example, individuals with stronger interpersonal skills may develop more supportive social networks, which are known to be associated with healthier behaviors and better stress resilience [23,32].

The results also have implications for health promotion. Although interventions targeting university students typically focus on individual behavior change, these findings suggest that developmental and family-context factors may also be relevant. Understanding the role of early family influences may help identify students who could benefit from additional support in developing coping strategies, self-regulation skills, and health responsibility. Furthermore, the findings may offer tentative support for further research exploring whether parenting-related educational or family-based preventive approaches are relevant in broader health promotion contexts [22]. From a broader public health perspective, these findings align with Sustainable Development Goal 3 (Good Health and Well-being), which emphasizes the promotion of healthy lives and well-being across the lifespan [33]. By identifying developmental and family-related correlates of health-promoting behaviors in young adulthood, the present study contributes to preventive health promotion efforts targeting student populations. Indirectly, fostering healthier and more resilient young adults may also support the broader aims of Sustainable Development Goal 11 (Sustainable Cities and Communities), particularly through strengthening community well-being and resilience [33], although this connection should be interpreted cautiously given the individual-level focus of the present study.

## 5. Limitations

Several limitations should be acknowledged. First, the cross-sectional design precludes causal inference, and associations should be interpreted cautiously. Second, both predictors and outcomes were assessed using self-report measures, introducing the possibility of recall bias, social desirability bias, and common-method bias, which may have inflated observed associations. Participants’ retrospective perceptions of parenting may also have been influenced by their current psychological functioning or health experiences. Third, although key demographic covariates were included in adjusted models, unmeasured confounding factors such as socioeconomic background, family structure, or personality characteristics cannot be excluded. Finally, the relatively low internal consistency of the permissive parenting subscale suggests reduced measurement precision for this construct, warranting cautious interpretation of findings involving permissive parenting.

## 6. Conclusions

The present study found significant associations between perceived parenting styles and health-promoting lifestyle behaviours among university students. Perceived authoritative parenting was consistently associated with more favorable outcomes across multiple health-promoting lifestyle domains, whereas permissive and authoritarian parenting showed weaker and less consistent associations. Given the cross-sectional design and reliance on retrospective self-reported perceptions, causal conclusions cannot be drawn. These findings contribute to understanding developmental and family-context correlates of health-related behaviours in young adulthood and may inform future longitudinal research.

## Figures and Tables

**Table 1:** Sample characteristics of the study participants (*N* = 701).

Characteristic	Category	*n* (%)
Gender	Men	194 (27.7)
	Women	497 (70.9)
	Other	5 (0.7)
	Prefer not to answer	4 (0.6)
Type of study	Full-time	675 (96.3)
	Part-time	25 (3.6)
Year of study	1st year	370 (52.8)
	2nd year	190 (27.1)
	3rd year	96 (13.7)
	4th year or higher	44 (6.3)
Employment alongside studies	Occasional job (e.g., student work)	402 (57.3)
	Regular employment	26 (3.7)
	No employment	272 (38.8)
Living arrangement	With parents	502 (71.6)
	With partner/family members	94 (13.4)
	Living alone	66 (9.4)
	Other	38 (5.4)
Chronic health condition	No	615 (87.7)
	Yes	85 (12.1)

**Table 2:** Descriptive characteristics and internal consistency of parenting style and health-promoting lifestyle measures (N = 700).

Variable	No. of Items	Mean	SD	Observed Range	Cronbach’s α
Authoritarian parenting	3	2.94	1.02	1.00–5.00	0.75
Authoritative parenting	3	3.83	0.76	1.00–5.00	0.64
Permissive parenting	3	2.83	0.74	1.00–5.00	0.48
Health Responsibility	8	1.95	0.49	1.00–4.00	0.77
Physical Activity	8	2.44	0.65	1.00–4.00	0.78
Nutrition	9	2.46	0.51	1.00–4.00	0.73
Spiritual Growth	9	2.84	0.62	1.00–4.00	0.86
Interpersonal Relations	9	2.97	0.59	1.00–4.00	0.86
Stress Management	8	2.58	0.59	1.00–4.00	0.75
HPLP II Total Score	51	2.55	0.40	1.00–3.94	0.92

**Table 3:** Zero-order correlations among parenting styles and health-promoting lifestyle variables (N = 700).

Variable	1	2	3	4	5	6	7	8	9	10
1. Authoritarian parenting	—									
2. Authoritative parenting	−0.12 **	—								
3. Permissive parenting	0.18 ***	−0.08 *	—							
4. Health Responsibility	−0.06	0.21 ***	−0.05	—						
5. Physical Activity	−0.03	0.24 ***	−0.11 **	0.42 ***	—					
6. Nutrition	−0.08 *	0.20 ***	−0.10 **	0.48 ***	0.44 ***	—				
7. Spiritual Growth	−0.14 ***	0.28 ***	−0.09 *	0.51 ***	0.39 ***	0.42 ***	—			
8. Interpersonal Relations	−0.16 ***	0.34 ***	−0.08 *	0.46 ***	0.35 ***	0.33 ***	0.62 ***	—		
9. Stress Management	−0.13 ***	0.26 ***	−0.09 *	0.49 ***	0.41 ***	0.39 ***	0.58 ***	0.60 ***	—	
10. HPLP II Total Score	−0.12 **	0.31 ***	−0.10 **	0.77 ***	0.73 ***	0.71 ***	0.84 ***	0.83 ***	0.80 ***	—

Note. * *p* < 0.05, ** *p* < 0.01, *** *p* < 0.001. HPLP II = Health-Promoting Lifestyle Profile II.

**Table 4:** Adjusted multiple linear regression examining associations between parenting styles and health-promoting lifestyle outcomes (N = 700).

Outcome	R^2^	Adj. R^2^	Authoritarian β (*p*)	Authoritative β (*p*)	Permissive β (*p*)
Health Responsibility	0.093	0.080	−0.054 (0.167)	0.190 (<0.001)	−0.006 (0.862)
Physical Activity	0.092	0.079	0.020 (0.613)	0.160 (<0.001)	−0.082 (0.027)
Nutrition	0.049	0.035	0.068 (0.090)	0.156 (<0.001)	−0.096 (0.011)
Spiritual Growth	0.129	0.116	−0.110 (0.004)	0.245 (<0.001)	−0.079 (0.030)
Interpersonal Relations	0.216	0.205	−0.119 (0.001)	0.309 (<0.001)	−0.045 (0.190)
Stress Management	0.072	0.059	−0.091 (0.021)	0.200 (<0.001)	−0.060 (0.108)
HPLP II Total Score	0.158	0.146	−0.072 (0.056)	0.305 (<0.001)	−0.090 (0.011)

**Table 5:** Multiple Linear Regression Predicting Health Responsibility (HPLP II) from Parenting Styles (N = 700).

Predictor	B	SE	β	t	*p*
Authoritarian	−0.01	0.02	−0.03	−0.69	0.490
Authoritative	0.14	0.02	0.23	6.04	<0.001
Permissive	−0.05	0.02	−0.08	−2.01	0.044
Constant	1.80	0.13	—	14.44	<0.001

Note. R^2^ = 0.062, adjusted R^2^ = 0.058. HR = Health Responsibility. Health Responsibility scores were calculated as the mean of the corresponding HPLP II items, with higher scores indicating greater engagement in health-responsible behaviors.

**Table 6:** Multiple Linear Regression Examining Associations Between Parenting Styles and Physical Activity (N = 700).

Predictor	B	SE	β	t	*p*
Authoritarian	−0.03	0.02	−0.06	−1.54	0.123
Authoritative	0.20	0.02	0.31	8.23	<0.001
Permissive	−0.06	0.02	−0.10	−2.64	0.008
Constant	2.11	0.13	—	16.02	<0.001

Note. R^2^ = 0.115, adjusted R^2^ = 0.111. PA = Physical Activity. Physical activity scores were calculated as the mean of the corresponding HPLP II items, with higher scores indicating more frequent engagement in physical activity.

**Table 7:** Multiple Linear Regression Predicting Nutrition (HPLP II) from Parenting Styles (N = 700).

Predictor	B	SE	β	t	*p*
Authoritarian	−0.04	0.02	−0.09	−2.29	0.022
Authoritative	0.15	0.02	0.25	6.50	<0.001
Permissive	−0.02	0.02	−0.03	−0.76	0.446
Constant	2.02	0.12	—	16.45	<0.001

Note. R^2^ = 0.083, adjusted R^2^ = 0.079. NU = Nutrition. Nutrition scores were calculated as the mean of the corresponding HPLP II items, with higher scores indicating more frequent engagement in healthy dietary behaviors.

**Table 8:** Multiple Linear Regression Predicting Spiritual Growth (HPLP II) from Parenting Styles (N = 700).

Predictor	B	SE	β	t	*p*
Authoritarian	−0.01	0.02	−0.02	−0.57	0.572
Authoritative	0.13	0.02	0.22	5.53	<0.001
Permissive	−0.04	0.02	−0.07	−1.79	0.074
Constant	2.11	0.13	—	16.42	<0.001

Note. R^2^ = 0.052, adjusted R^2^ = 0.048. SG = Spiritual Growth. Spiritual growth scores were calculated as the mean of the corresponding HPLP II items, with higher scores indicating higher levels of spiritual growth.

**Table 9:** Multiple Linear Regression Predicting Interpersonal Relations (HPLP II) from Parenting Styles (N = 700).

Predictor	B	SE	β	t	*p*
Authoritarian	−0.03	0.02	−0.07	−1.80	0.072
Authoritative	0.21	0.02	0.33	8.84	<0.001
Permissive	−0.08	0.02	−0.12	−3.36	<0.001
Constant	2.26	0.13	—	17.55	<0.001

Note. R^2^ = 0.135, adjusted R^2^ = 0.131. IR = Interpersonal Relations. The IR subscale was calculated using eight items, as one item from the original HPLP II instrument was not included in the questionnaire. Higher scores indicate better interpersonal relations.

**Table 10:** Multiple Linear Regression Predicting Stress Management (HPLP II) from Parenting Styles (N = 700).

Predictor	B	SE	β	t	*p*
Authoritarian	−0.04	0.02	−0.08	−2.08	0.038
Authoritative	0.16	0.03	0.24	6.21	<0.001
Permissive	−0.08	0.03	−0.11	−2.96	0.003
Constant	2.49	0.14	—	17.98	<0.001

Note. R^2^ = 0.083, adjusted R^2^ = 0.079. SM = Stress Management. Stress management scores were calculated as the mean of the corresponding HPLP II items, with higher scores indicating more effective stress management behaviors.

**Table 11:** Multiple Linear Regression Predicting Overall Health-Promoting Lifestyle (HPLP II Total Score) from Parenting Styles (N = 700).

Predictor	B	SE	β	t	*p*
Authoritarian	−0.03	0.02	−0.07	−1.85	0.064
Authoritative	0.16	0.02	0.31	8.25	<0.001
Permissive	−0.05	0.02	−0.09	−2.59	0.010
Constant	2.14	0.11	—	19.88	<0.001

Note. R^2^ = 0.118, adjusted R^2^ = 0.115. The HPLP II total score was calculated as the mean of all available HPLP II items, with higher scores indicating a more health-promoting lifestyle.

**Table 12:** Adjusted Multiple Linear Regression Examining Associations Between Parenting Styles and Health Responsibility (HPLP II) (N = 700).

Outcome	R^2^	Adj. R^2^	Authoritarian β	Authoritative β	Permissive β
Health Responsibility	0.093	0.080	−0.054, *p* = 0.167	0.190, *p* < 0.001	−0.006, *p* = 0.862
Physical Activity	0.092	0.079	0.020, *p* = 0.613	0.160, *p* < 0.001	−0.082, *p* = 0.027
Nutrition	0.049	0.035	0.068, *p* = 0.090	0.156, *p* < 0.001	−0.096, *p* = 0.011
Spiritual Growth	0.129	0.116	−0.110, *p* = 0.004	0.245, *p* < 0.001	−0.079, *p* = 0.030
Interpersonal Relations	0.216	0.205	−0.119, *p* = 0.001	0.309, *p* < 0.001	−0.045, *p* = 0.190
Stress Management	0.072	0.059	−0.091, *p* = 0.021	0.200, *p* < 0.001	−0.060, *p* = 0.108
HPLP II Total	0.158	0.146	−0.072, *p* = 0.056	0.305, *p* < 0.001	−0.090, *p* = 0.011

## Data Availability

The data presented in this study are available on request from the corresponding author. Data are restricted due to ethical and privacy considerations, as they contain sensitive information that could potentially compromise the confidentiality of research participants.
